# Artificial Intelligence in Bulk RNA-Seq: Challenges and Potential Solutions

**DOI:** 10.34133/csbj.0039

**Published:** 2026-04-01

**Authors:** Mostafa Rezapour, Stephanie V. Trefry, Lorreta A. Opoku, Aarthi Narayanan

**Affiliations:** ^1^ Wake Forest Institute for Regenerative Medicine, Wake Forest University School of Medicine, Winston-Salem, NC 27101, USA.; ^2^ Department of Biology, College of Science, George Mason University, Fairfax, VA 22030, USA.; ^3^ School of Systems Biology, College of Science, George Mason University, Fairfax, VA 22030, USA.

## Abstract

Bulk RNA sequencing (RNA-seq) produces high-dimensional gene expression data where the number of measured features greatly exceeds the number of available samples, which challenges artificial intelligence (AI)-based modeling. In this setting, models are highly susceptible to overfitting and may fail to generalize across independent datasets when feature dimensionality is not adequately controlled. Feature (gene) selection is therefore essential for reliable inference, particularly when it is performed strictly within training data to prevent information leakage. This review examines how high dimensionality and limited sample size constrain AI-based analysis of bulk RNA-seq data and surveys feature selection strategies used to address these challenges. Emphasis is placed on statistically guided, training-only frameworks, with a focus on generalized linear models with quasi-likelihood *F* tests and magnitude–altitude scoring (GLMQL-MAS). Across published viral infection studies, GLMQL-MAS yields compact, interpretable gene sets that support robustness, reproducibility, and cross-dataset generalization in bulk transcriptomic modeling.

## Introduction

Bulk RNA sequencing (RNA-seq) enables genome-wide quantification of transcript abundance, generating expression measurements for tens of thousands of genes per sample. These datasets are intrinsically high dimensional, where the number of molecular features far exceeds the number of biological samples typically available. Such conditions place bulk RNA-seq data firmly within the setting of the curse of dimensionality [1,2], where expansion of feature space without proportional sample support limits model generalizability.

In practice, bulk RNA-seq studies are frequently constrained by limited sample size due to cost, experimental complexity, or restricted availability of biological material. Analytical settings defined by small sample size and large feature dimensionality, where features correspond to measured transcripts or genes, are particularly susceptible to overfitting, where models learn dataset-specific noise rather than reproducible biological signal [3]. Models affected by overfitting often exhibit high apparent performance during training yet fail to generalize to independent datasets, which undermines reproducibility and translational relevance.

**Fig. 1.**
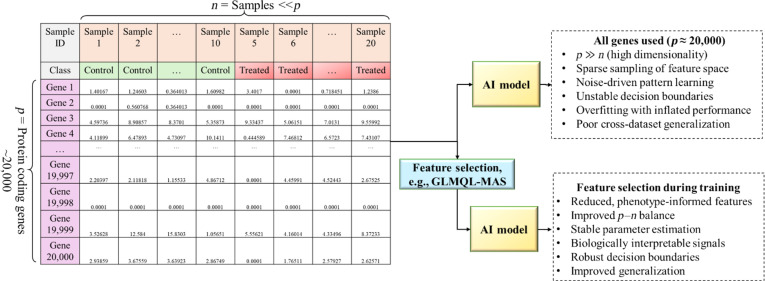
High dimensionality of bulk RNA-seq data and the role of training-only feature selection in AI-based modeling. Bulk RNA-seq data comprise approximately 20,000 protein-coding genes per sample, which creates a high-dimensional learning setting with elevated overfitting risk under limited sample size. Statistically guided feature selection is applied strictly within the training data, either prior to cross-validation or independently within each training fold, to reduce dimensionality while preventing information leakage. The resulting reduced gene set supports more stable model fitting and improved generalization to held-out data.

Feature selection provides a principled strategy to mitigate overfitting by restricting the predictor space to genes that show statistically supported associations with phenotypic outcomes. When feature selection is performed exclusively within training data, it reduces noise while preserving strict separation between model development and evaluation (Fig. 1). For this reason, statistically guided gene pre-selection can be used as a foundational component of predictive modeling pipelines in bulk RNA-seq studies, particularly under small-sample conditions.

Among available feature selection frameworks, generalized linear models with quasi-likelihood *F* tests and magnitude–altitude scoring (GLMQL-MAS) [4–10] offers a statistically rigorous and phenotype-informed approach for identifying informative transcriptomic features. By combining edgeR-based [11] generalized linear modeling with composite ranking that integrates effect size and statistical confidence, GLMQL-MAS produces compact gene sets that support robust and reproducible downstream modeling.

This review examines the interaction between high dimensionality and limited sample size in bulk RNA-seq data, the implications for artificial intelligence (AI)-based modeling, and the role of statistically grounded, training-only feature selection frameworks such as GLMQL-MAS in addressing overfitting and reproducibility limitations.

## AI in Bulk Transcriptomics: High Dimensionality, Overfitting, and the Role of Feature Selection

AI methods have become widely adopted in bulk RNA-seq analysis due to their capacity to model complex, multivariate relationships across large gene expression spaces [12]. However, their direct application to bulk transcriptomic data is fundamentally constrained by the structural properties of these datasets. Bulk RNA-seq studies typically measure expression for tens of thousands of genes while containing only a limited number of samples, which places AI models in a setting where reliable parameter estimation is difficult and predictive performance often fails to generalize beyond the training data.

Most machine learning models process all provided input features without any inherent mechanism to distinguish biologically meaningful signal from noise unless explicit feature selection or regularization components are introduced. When bulk RNA-seq expression matrices are used without prior feature restriction, models are exposed to thousands of genes, many of which exhibit low expression, weak phenotype association, or stochastic variation. Under small-sample conditions, this configuration substantially increases the likelihood that models assign importance to noise-driven features, which results in overfitting and inflated training performance.

High-capacity learning architectures, including ensemble methods [13] and deep neural networks [14], are particularly vulnerable in this setting when model complexity is not matched to sample size and feature dimensionality. Their flexibility enables close fitting of training data, which in high-dimensional transcriptomic spaces often leads to memorization of dataset-specific artifacts rather than extraction of reproducible biological signal. As a consequence, learned patterns may reflect technical variation or cohort-specific structure instead of phenotype-associated transcriptional programs.

Overfitting in AI-based bulk RNA-seq analysis often arises from a fundamental mismatch between data dimensionality and sample availability rather than model choice alone. Without statistically grounded feature selection applied strictly within training data, and without matching model complexity to the number of available samples and selected features, AI models may remain prone to learning noise and technical artifacts.

To address these challenges, feature selection strategies used in transcriptomic machine learning must balance statistical rigor, compatibility with the distributional properties of RNA-seq data, and preservation of biological interpretability. Approaches differ in how they incorporate phenotype information, how they rank candidate genes, and whether they retain genes as interpretable predictors or transform the data into latent representations. These methodological differences influence the stability of selected gene sets and the reliability of downstream predictive models. The following section therefore examines GLMQL-MAS in detail and places it within the broader landscape of feature selection approaches used in bulk RNA-seq analysis, with emphasis on how its statistical design supports interpretable and robust gene selection for predictive modeling.

## GLMQL-MAS: A Statistically Grounded Framework for Feature Selection in Bulk RNA-seq Analysis

The principal approaches that can be used to control dimensionality in transcriptomic machine learning include statistical gene-ranking methods, machine learning-based feature selection strategies, probabilistic variable selection frameworks, dimensionality reduction techniques, and neural-network regularization strategies. Each category provides different mechanisms for identifying relevant predictors and mitigating overfitting. However, these approaches also differ in their compatibility with the statistical properties of bulk RNA-seq data and in the extent to which they preserve biological interpretability.

### Statistical gene-ranking approaches

Many transcriptomic machine learning workflows begin by ranking genes using differential expression analysis and selecting the highest-ranked genes as predictors for downstream classifiers. Common frameworks include DESeq2 [15] and limma-voom [16], which evaluate gene–phenotype associations using statistical models that account for experimental design and biological variability. Genes are then prioritized according to metrics such as *P* values, adjusted *P* values, or *log_2_* fold change. This strategy provides a straightforward mechanism for dimensionality reduction while maintaining a clear statistical connection between gene expression and phenotype association.

Univariate filtering methods such as the Fisher score [17] similarly can evaluate genes independently by comparing between-class and within-class variance. While computationally efficient, these methods do not account for gene–gene dependencies and do not incorporate statistical models tailored to RNA-seq count data. As a result, their rankings may be sensitive to technical variation and may not fully capture the distributional properties of transcriptomic measurements.

### Machine learning feature selection methods

Machine learning feature selection approaches identify subsets of genes that improve predictive performance of downstream classifiers. These methods incorporate phenotype information during feature selection and often consider relationships among predictors.

Wrapper-based approaches such as minimum redundancy maximum relevance (mRMR) [18] can select genes that maximize relevance to the phenotype while minimizing redundancy among selected features. This strategy attempts to identify compact gene sets that contain complementary predictive information. However, reliable estimation of mutual information requires sufficient sample size, which is often limited in bulk RNA-seq studies.

Embedded feature selection methods integrate gene selection directly into model training. Lasso regression [19] performs variable selection by imposing an L1 penalty that drives some coefficients to zero, which yields sparse predictive models. Elastic Net regression [20] extends this strategy by combining L1 and L2 penalties, which improves stability when predictors are highly correlated and allows groups of correlated genes to be retained.

Boruta feature importance [21] uses random forest (RF) classifiers to estimate feature importance and compares observed gene importance to randomized shadow features that provide an empirical baseline. Genes that consistently outperform shadow features are retained as relevant predictors. Although Boruta can detect nonlinear interactions, its importance estimates may be sensitive to correlation structure and stochastic variation, particularly when sample size is limited.

Another strategy for improving robustness of selected features is stability selection [22]. In this framework, regularized models such as Lasso or Elastic Net are repeatedly fitted on subsampled versions of the training dataset, and genes that are consistently selected across multiple resampled models are retained as stable predictors. This approach reduces sensitivity to sampling variability but remains dependent on the assumptions of the underlying regression model.

### Probabilistic variable selection approaches

Probabilistic feature selection methods model uncertainty in feature inclusion directly. Bayesian variable selection frameworks [23] introduce prior distributions over model parameters and estimate posterior probabilities that individual genes contribute to predictive models.

By explicitly modeling uncertainty in feature inclusion, Bayesian approaches can incorporate prior biological knowledge and generate probabilistic rankings of candidate genes. However, Bayesian variable selection methods can become computationally demanding when applied to genome-scale datasets containing tens of thousands of predictors. Furthermore, many implementations rely on transformed expression measurements rather than directly modeling RNA-seq count distributions.

### Dimensionality reduction and representation learning

Dimensionality reduction techniques address high dimensionality by transforming gene expression data into lower-dimensional representations. Projection-based approaches such as principal components analysis (PCA) [24] and independent components analysis (ICA) [25] generate latent components that summarize patterns of variation across genes. These components can then be used as predictors for downstream modeling tasks.

Deep learning methods extend this concept through representation learning. Autoencoders compress the input gene expression matrix into a lower-dimensional latent space that captures major structure in the data before reconstructing the original input [26]. Related latent embedding approaches generate compact representations suitable for predictive modeling [27]. Although these approaches can capture complex relationships among genes, the resulting latent variables represent combinations of many genes and therefore reduce direct biological interpretability compared with gene-level feature selection.

### Neural-network regularization strategies

Deep learning models often address high dimensionality through regularization techniques that constrain model flexibility during training rather than selecting genes explicitly. Dropout randomly omits a subset of hidden units during each training iteration, which reduces co-adaptation among neurons and improves generalization [28]. Early stopping halts training when validation performance ceases to improve, which prevents continued fitting to noise in the training data [3]. Weight decay, typically implemented as L2 regularization, penalizes large parameter values and encourages smaller weight magnitudes that generalize more reliably [29]. These strategies reduce effective model complexity but do not directly identify biologically interpretable gene subsets.

### GLMQL-MAS framework

Although the methods described above provide various strategies for dimensionality control, many approaches either rely on statistical assumptions that do not explicitly reflect the discrete and overdispersed structure of RNA-seq count data or transform gene expression measurements into latent variables that reduce biological interpretability. In addition, several gene-ranking strategies prioritize genes using a single selection criterion, such as statistical significance (*P* value) or expression effect size (*log_2_* fold change), which may not fully capture multiple aspects of gene relevance. GLMQL-MAS [4–10] was developed to address these limitations through a statistically grounded feature selection framework designed specifically for bulk RNA-seq predictive modeling.

Unlike projection-based dimensionality reduction methods, GLMQL-MAS retains individual genes as predictors. This design preserves biological interpretability and enables direct linkage between selected genes and disease-related molecular mechanisms.

The statistical foundation of GLMQL-MAS is based on generalized linear modeling of RNA-seq count data using the negative binomial distribution implemented in edgeR [11]. This framework explicitly models the discrete and overdispersed nature of RNA-seq measurements, where variance frequently exceeds the mean [15,30]. Raw read counts are normalized using the trimmed mean of *M* values method [31] to correct for library size differences and compositional bias. Experimental conditions and covariates are encoded through a design matrix, and gene-wise dispersion estimates capture inter-sample variability.

Gene–phenotype associations are evaluated using quasi-likelihood *F* tests [32], which account for uncertainty in dispersion estimation and improve control of false positives in small-sample settings. Resulting *P* values are adjusted for multiple testing using the Benjamini–Hochberg procedure [33], yielding statistically controlled significance estimates for each gene.

To prioritize genes by combined biological effect size and statistical confidence, GLMQL-MAS applies magnitude–altitude scoring (MAS) [34,35], which integrates absolute *log_2_* fold change and adjusted *P* value into a composite ranking metric. For each significant gene *l*, a composite MAS score is computed as follows:

$${\mathrm{MAS}}_{l}={\left|\;\log_{2}\left({\mathrm{FC}}_{l}\right)\right|}^{M}{\left|\log_{10}\left(P_{l}^{\mathrm{BH}}\right)\right|}^{A}$$ (1)

where $P_{l}^{\mathrm{BH}}$ denotes the BH adjusted *P* value and ${\mathrm{FC}}_{l}$ is the fold change. This approach avoids reliance on a single selection criterion and favors genes that exhibit both large absolute expression changes and strong statistical support, with weighting controlled by the user-defined parameters M and A. When the raw *P* value $p_{l}$ is used instead of $P_{l}^{\mathrm{BH}}$ in Eq. (1), the resulting metric is termed the relaxed magnitude–altitude score (RMAS) [4], and the associated pipeline is referred to as GLMQL-RMAS. For multi-condition experimental designs, Cross-MAS [6] integrates rankings across contrasts, promoting genes with consistent effects while preserving condition-specific signals.

**Fig. 2.**
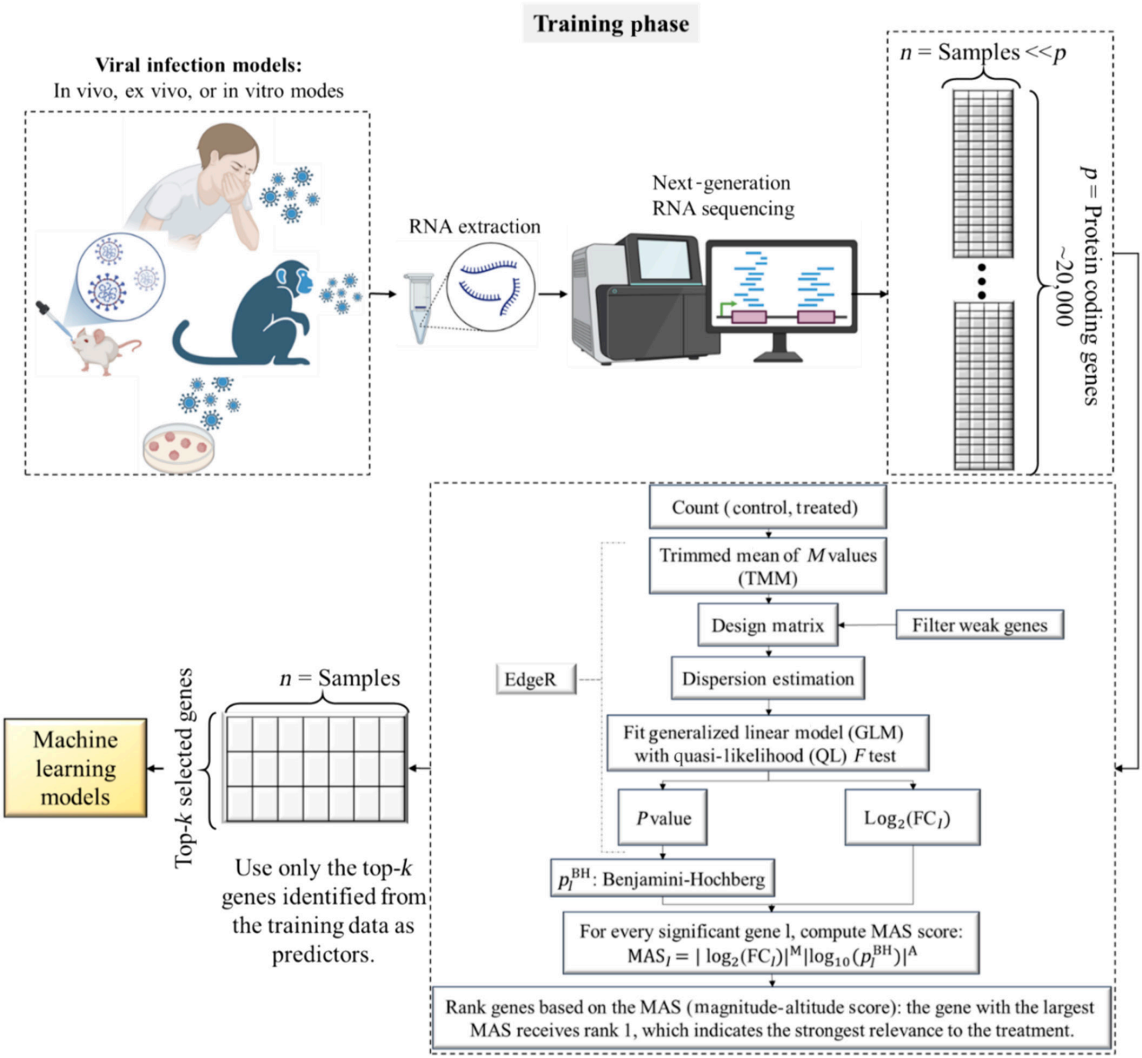
Schematic overview of the GLMQL-MAS training-phase feature selection framework for bulk RNA-seq analysis. The schematic illustrates normalization, dispersion modeling, quasi-likelihood testing, and magnitude–altitude scoring used to rank genes within training data, yielding compact and interpretable gene signatures for downstream machine learning.

**Fig. 3.**
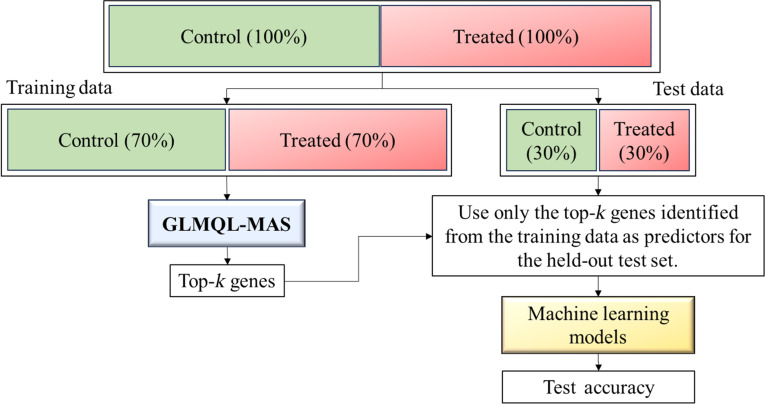
Dataset splitting and training-only feature selection using GLMQL-MAS. GLMQL-MAS is applied exclusively to training data to identify top-*k* gene signatures, which are then used for machine learning and evaluated on held-out test data.

Figure 2 presents a schematic overview of the GLMQL-MAS pipeline, which illustrates normalization, dispersion modeling, quasi-likelihood testing, and MAS-based gene ranking performed strictly within the training phase. Figure 3 further illustrates the separation of training and held-out test datasets required for valid performance evaluation, with GLMQL-MAS applied exclusively to the training data to identify gene signatures subsequently evaluated on independent samples.

### Comparative assessment of feature selection strategies for bulk RNA-seq predictive modeling

Taken together, the feature selection and dimensionality control strategies outlined in Statistical gene-ranking approaches, Machine learning feature selection methods, Probabilistic variable selection approaches, Dimensionality reduction and representation learning, and Neural-network regularization strategies sections address high-dimensional transcriptomic data through distinct mechanisms, including univariate statistical ranking, predictive feature subset selection, probabilistic variable inclusion, latent representation learning, and model regularization. In contrast, GLMQL-MAS provides a statistically grounded feature selection framework that directly models RNA-seq count data and prioritizes genes using combined measures of statistical significance and expression effect size. The suitability of these approaches for bulk RNA-seq analysis therefore depends on whether they preserve gene-level interpretability, accommodate limited sample size, and appropriately account for the discrete and overdispersed structure of RNA-seq measurements.

Statistical gene-ranking approaches such as DESeq2 [15], limma-voom [16], and univariate filtering methods such as the Fisher score [17] provide interpretable gene-level prioritization and can be used for dimensionality reduction prior to machine learning. However, gene ranking in these pipelines is often based on a single criterion, such as statistical significance or fold change, which may not fully capture multiple aspects of gene relevance. Machine learning feature selection methods including mRMR [18], Lasso regression [19], Elastic Net [20], and Boruta feature importance [21] can identify compact predictive subsets and may capture dependencies among predictors. Nevertheless, these approaches typically operate on transformed expression values and do not explicitly model RNA-seq count distributions or overdispersion.

Probabilistic variable selection frameworks such as Bayesian feature selection [23] incorporate uncertainty in feature inclusion through posterior probability estimates, although their computational demands may limit application to genome-scale transcriptomic datasets. Dimensionality reduction and representation learning approaches, including PCA [24], ICA [25], and neural autoencoder models [26], compress gene expression data into latent representations that summarize global expression structure. While effective for reducing dimensionality, these approaches replace individual genes with latent variables and therefore reduce direct biological interpretability.

Finally, neural-network regularization strategies such as dropout [28], early stopping [3], and weight decay [29] improve model generalization by constraining parameter learning during training, but they do not directly identify informative gene subsets. Moreover, these techniques are typically applied within deep learning architectures that contain large numbers of trainable parameters and therefore require sufficiently large training datasets to achieve stable model estimation. In bulk RNA-seq studies, where the number of measured genes often greatly exceeds the number of available samples, such models may remain prone to overfitting despite regularization and do not directly identify interpretable gene subsets.

Within this landscape, GLMQL-MAS differs in that it combines RNA-seq-appropriate count modeling, quasi-likelihood-based inference, effect-size-aware ranking, and strict training-only feature selection while retaining genes as interpretable predictors. This combination is particularly relevant in bulk RNA-seq studies with limited sample size, where robustness, biological interpretability, and protection against information leakage are central considerations. Rather than relying on latent representations or feature selection procedures detached from count-based statistical inference, GLMQL-MAS integrates gene prioritization within a framework specifically matched to the statistical properties of bulk RNA-seq data. Table 1 provides a comprehensive comparison of these methods and highlights the conceptual and practical characteristics that distinguish GLMQL-MAS from alternative strategies for dimensionality control in transcriptomic predictive modeling.

**Table 1.** Comparison of GLMQL-MAS and classical feature selection and dimensionality reduction methods in bulk RNA-seq analysis. Methods marked with an asterisk select predictors that correspond to genes, but the stability of the selected gene set may be affected under conditions of strong feature correlation or very small sample sizes.

Method	Category	Models RNA-seq count distribution	Accounts for overdispersion	Produces interpretable genes	Interpretability of reduced dimension	Gene selection/ranking criterion
Fisher score	Statistical ranking	No	No	Yes	Ranked gene list	Between-class vs. within-class variance
DESeq2/limma-voom ranking	Statistical ranking/differential expression filter	Yes	Yes	Yes	Ranked gene list	Differential expression significance (*P* value, adjusted *P* value, or log_2_FC)
mRMR	Filter feature selection	No	No	Yes	Selected gene subset	Maximum relevance to phenotype and minimum redundancy among genes
Lasso regression	Embedded feature selection	No	No	Yes*	Selected gene subset	L1-regularized model coefficients
Elastic Net	Embedded feature selection	No	No	Yes*	Selected gene subset	Combined L1 and L2 regularized coefficients
Boruta	Wrapper feature selection	No	No	Yes	Selected gene subset	Random forest feature importance relative to shadow features
Stability selection	Resampling-based feature selection	No	No	Yes*	Selected gene subset	Selection frequency across resampled models
Bayesian variable selection	Probabilistic selection	No	No	Yes	Selected gene subset	Posterior inclusion probability
PCA	Dimensionality reduction	No	No	No	Latent components	Variance-maximizing principal components
ICA	Dimensionality reduction	No	No	No	Latent components	Statistical independence of latent components
Autoencoder	Representation learning	No	No	No	Latent embeddings	Reconstruction-based latent representation learning
Deep learning regularization	Model regularization	No	No	No	Model parameters	Parameter regularization (dropout, weight decay, early stopping)
GLMQL-MAS	Statistical ranking/differential expression filter	Yes	Yes	Yes	Ranked gene list	Integrated adjusted *P* value and log_2_ fold change (magnitude–altitude score)

Taken together, the comparison summarized in Table 1 highlights that GLMQL-MAS is among the approaches that combine RNA-seq-appropriate statistical modeling, phenotype-guided inference, effect-size-aware ranking, and strict training-only feature selection. In this pipeline, GLMQL-MAS explicitly integrates these components within a single training-only feature selection module while retaining genes as interpretable features. A recent comparative evaluation of GLMQL-MAS variants demonstrated improved performance relative to some alternative methods [10].

Therefore, GLMQL-MAS functions as an upstream feature selection module that yields compact, biologically interpretable gene sets for downstream classification, where model complexity is matched to sample size to reduce overfitting and support reproducible transcriptomic modeling. The selected genes serve as predictors for supervised learning, with linear classifiers such as logistic regression [36] or linear support vector machines [37] favored under small-sample conditions due to their reliable generalization. With larger cohorts, tree-based ensemble methods, including RFs and boosting approaches [38], can be applied to capture nonlinear effects and interactions.

## Applications of GLMQL-MAS in Bulk Transcriptomics of Viral Infections

Across diverse biological contexts, GLMQL-MAS and related magnitude–altitude frameworks have been applied to bulk RNA-seq studies that are high dimensional and small in sample size, which makes robust, statistically guided feature selection central for reproducible modeling. In this review, we focus specifically on its use in viral infection studies.

In upper-airway lung organ tissue equivalents infected with influenza A virus, human metapneumovirus, or parainfluenza virus type 3, GLMQL-MAS highlighted a conserved innate antiviral program enriched for interferon-stimulated genes, including *IFIT1*, *IFIT2*, *IFIT3*, and *OAS* family members, and supported compact signatures that improved multinomial classification under stratified cross-validation [4]. In human induced pluripotent stem cell-derived colon organoids infected with multiple Mpox clades, GLMQL-RMAS and Cross-RMAS identified clade-discriminatory stress and epithelial-response genes, and the resulting compact set enabled clear separation under strict cross-validation, which demonstrated stable feature discovery under severe sample constraints [6].

Cross-platform robustness was demonstrated in Ebola virus studies, where *OAS1* was selected and served as a single-gene predictor that achieved clear discrimination across platforms and an independent held-out human dendritic cell dataset [7]. Variant-agnostic signatures for SARS-CoV-2 were derived via GLMQL-MAS and Cross-MAS across multiple cohorts, which produced conserved ranked genes that included *IFI27*, *CDC20*, *RRM2*, *HJURP*, *CDC45*, and *BIRC5* and supported high accuracy across held-out blood datasets plus external tissues and stimulation systems [8]. Finally, in primary human endothelial cells infected with Venezuelan equine encephalitis virus (VEEV), GLMQL-MAS and Cross-MAS separated an interferon and cytokine-driven untreated response from an NRF2-centered omaveloxolone program while preserving a shared antiviral backbone, which provided mechanistic stratification without relying on supervised classification [39].

Across the diverse viral systems summarized in Table 2, GLMQL-MAS-based feature selection consistently prioritizes a conserved host antiviral transcriptional core, which emerges independently across tissues, species, and experimental platforms. This convergence reflects the fact that interferon-stimulated genes exhibit larger and more consistent infection-associated expression changes than most other transcripts in bulk RNA-seq data, which makes them statistically prominent under rigorous modeling of count-based variance.

**Table 2.** Applications of GLMQL-MAS in bulk transcriptomic studies of viral infections, summarizing host systems, viruses analyzed, selected gene signatures, downstream modeling approaches, and reported performance metrics

Host and system	Virus or viruses	GLMQL-MAS selected genes	Machine learning model	Performance metrics
Human lung upper-airway organ tissue equivalents (OTEs), in vitro air–liquid interface model derived from non-diseased human lung donor tissue [4]	Influenza A virus (IAV, H1N1 PR8), human metapneumovirus (MPV), parainfluenza virus type 3 (PIV3)	*IFIT1* and *IFIT2* as infection-dependent predictors; *ELOVL4* as a time-dependent predictor. The final classifier used these 3 genes	Multinomial logistic regression for 8-class classification (3 viruses × 2 time points plus Mock-24 and Mock-72), 6 biological replicates per class (48 samples total), evaluated using 6-fold stratified cross-validation	Mean accuracy 92% (95% CI: 85–98%) across cross-validation folds
Human induced pluripotent stem cell-derived colon organoids, in vitro model of human gastrointestinal epithelium [6]	Mpox virus (MPXV) clade I (Zr-599, Congo Basin), clade IIa (Liberia), clade IIb (2022 outbreak strain)	Cross-RMAS identified clade-discriminatory genes including *TFF1*, *HSPA6*, *FOS*, *GSTA5*, *MPIG6B*, *F2*, *HP*, with additional Cross-MAS genes *DUSP1* and *SERPINA3*	Logistic regression and support vector machine with linear kernel using one-versus-one and one-versus-rest strategies; 12 samples total, evaluated using leave-one-out or 3-fold stratified cross-validation	100% accuracy with 100% macro-precision, recall, and F1-score under cross-validation
Cynomolgus macaques (non-human primates), in vivo whole blood for training and validation; human monocyte-derived dendritic cells, in vitro for held-out testing [7]	Ebola virus (EBOV/Makona in NHPs; wild-type, VP35m, VP24m, and VP35m + VP24m mutants in human DCs)	*OAS1* identified as the top GLMQL-MAS ranked gene; additional prioritized genes included *ISG15*, *IFI44*, *IFI27*, *IFIT2*, *IFIT3*, *IFI44L*, *MX1*, *MX2*, *OAS2*, *RSAD2*, and *OASL*	Logistic regression using a single-gene predictor (*OAS1*) to reduce overfitting; trained and validated on RNA-seq data from 12 EBOV-infected cynomolgus macaques using 6-fold stratified cross-validation, with evaluation on an independent held-out RNA-seq dataset of EBOV-infected human dendritic cells	100% accuracy in distinguishing EBOV-infected from non-infected samples in NHP RNA-seq data under cross-validation, and 100% accuracy on the independent held-out human DC dataset, confirmed using 1,000 bootstrap evaluations
Human blood-derived transcriptomes from public cohorts (whole blood, white blood cells, PBMCs) with COVID-negative controls; plus held-out validation in independent blood/PBMC cohorts and additional non-blood contexts [8]	SARS-CoV-2 variants including Original Wuhan, French cohort cases, Beta, and Omicron (training cohorts from GEO: GSE157103, GSE171110, GSE189039, GSE201530); held-out test cohorts included GSE152418, PMC8202013, GSE161731, GSE166190; additional datasets included GSE294888 (pDC/DC2 stimulation with Delta or Omicron) and GSE239595 (Omicron-infected nasopharyngeal tissue)	Cross-MAS selected a variant-agnostic signature set that included top-ranked genes such as *IFI27*, *CDC20*, *RRM2*, *HJURP*, and *CDC45* (with an expanded Cross-MAS list used downstream that included additional genes, which include *BIRC5* and other cell-cycle and ribosomal markers)	Logistic regression and linear SVM using principal components derived from Cross-MAS selected genes, trained on 4 RNA-seq blood/PBMC cohorts (199 samples total) and evaluated on independent held-out datasets comprising 211 samples across 4 cohorts	97.31% accuracy on aggregated held-out blood/PBMC test datasets, with precision 0.97 and recall 0.99. Classification was reported in 2 additional datasets, which included 100% accuracy, precision, and recall in GSE294888 and GSE239595
Primary human umbilical vein endothelial cells (HUVECs), in vitro [39]	Venezuelan equine encephalitis virus (VEEV), TC-83 strain	GLMQL-MAS prioritized an interferon-stimulated antiviral core including *IFIT1*, *IFIT2*, *IFIT3*, *OASL*, *RSAD2*, *MX1*, *IL6*, *CXCL10*, and *CXCL11*. Under omaveloxolone treatment, Cross-MAS identified an NRF2-centered signature dominated by *HMOX1*, *NQO1*, *GCLM*, *TXNRD1*, *SLC7A11*, *FTL*, and *FTH1*, with a compact 34-gene shared antiviral backbone	GLMQL-MAS and Cross-MAS were used for feature prioritization, stratification, clustering, and network-level analysis	Focus was on mechanistic transcriptional reprogramming and pathway-level discrimination

Note that several studies reported perfect separation; however, such results may reflect highly discriminative signals and modest sample sizes in specific experimental settings, and performance may be lower in larger or more heterogeneous cohorts.

## Application Scenario Boundaries

Although GLMQL-MAS provides a statistically grounded framework for training-only feature selection in bulk RNA-seq analysis, its applicability depends on several characteristics of the input dataset and study design. As with other statistical modeling approaches applied to high-dimensional transcriptomic data, the reliability of gene prioritization can be influenced by factors such as sample size, technical variation, experimental complexity, and the nature of the phenotype being modeled.

In particular, extremely small sample sizes can limit the stability of dispersion estimation and gene-level inference within generalized linear models. When the number of biological replicates per condition falls below approximately 5 samples, statistical power becomes severely constrained and gene rankings derived from GLMQL-MAS may become sensitive to individual sample variability. Under such conditions, the resulting feature sets may reflect stochastic variation rather than stable phenotype-associated transcriptional signals. In these settings, results obtained with GLMQL-MAS should therefore be interpreted cautiously and ideally validated using independent datasets or complementary experimental evidence.

Strong batch effects represent another scenario that may affect the reliability of statistically guided feature selection. Bulk RNA-seq datasets that combine samples from different sequencing runs, laboratories, or experimental protocols can contain systematic technical variation unrelated to the biological phenotype of interest. If such effects are not properly accounted for within the design matrix or through appropriate preprocessing procedures, batch-associated variation may influence gene ranking and lead to the prioritization of technically driven features. Standard RNA-seq workflows typically address this issue through explicit modeling of batch covariates or through normalization and correction procedures prior to differential expression analysis.

Experimental designs that include multiple biological factors or covariates can also influence the interpretation of gene prioritization results. Although the generalized linear modeling framework underlying GLMQL-MAS supports complex design matrices that incorporate multiple variables, gene ranking may depend on the contrasts used to define phenotype associations. In studies involving several interacting experimental factors, careful specification of contrasts and covariate structure is therefore important to ensure that prioritized genes reflect the biological processes of interest rather than secondary sources of variation.

The nature of the phenotype being modeled may also affect the behavior of statistically guided feature selection. While many bulk RNA-seq studies involve categorical phenotypes such as disease versus control or infected versus uninfected conditions, some applications involve continuous phenotypic variables such as biomarker levels, clinical severity scores, or viral load measurements. In such cases, generalized linear modeling can incorporate quantitative predictors within the design matrix; however, the stability of gene ranking may depend on the strength of the association signal and the available sample size.

Finally, the relative advantages of different feature selection strategies may vary across dataset scales. GLMQL-MAS is particularly well suited for high-dimensional transcriptomic datasets with limited sample size, where robust statistical modeling of RNA-seq count distributions and strict training-only feature selection can help reduce overfitting and preserve biological interpretability. In contrast, in larger cohort settings that include hundreds or thousands of samples, embedded machine learning approaches such as ensemble-based feature selection methods may become increasingly competitive for predictive modeling tasks. In such scenarios, statistically guided gene prioritization approaches such as GLMQL-MAS may function as complementary strategies for interpretable feature selection rather than the sole dimensionality control mechanism.

Taken together, GLMQL-MAS is most reliable when applied to datasets that contain adequate biological replication and where major sources of technical variation are either controlled experimentally or explicitly modeled during statistical analysis. Within these conditions, the framework provides stable gene prioritization and compact feature sets suitable for downstream machine learning while preserving gene-level interpretability and alignment with the statistical properties of bulk RNA-seq data.

## Conclusions

Bulk RNA-seq analysis operates at the intersection of extreme feature dimensionality and limited sample size, which creates a structural propensity for overfitting and poor generalization in AI-based modeling. Across this review, evidence from published studies indicates that these limitations arise less from model choice and more from inadequate control of the feature space. Feature selection that is statistically grounded, phenotype informed, and confined strictly to training data emerges as a necessary prerequisite for reliable inference, reproducibility, and cross-dataset transfer in bulk transcriptomics.

GLMQL-MAS addresses these challenges by aligning feature selection with the statistical properties of RNA-seq count data and by integrating effect size with controlled inference. Its reliance on generalized linear modeling with quasi-likelihood testing enables robust gene-level prioritization under small-sample conditions, while magnitude–altitude scoring produces compact and interpretable gene sets that reduce noise without discarding biologically meaningful signal. Across viral infection studies spanning tissues, platforms, and viral families, GLMQL-MAS consistently converges on conserved interferon-driven host response programs and supports models that generalize beyond their training cohorts. These applications demonstrate that statistically guided, training-only feature selection can stabilize AI workflows and reveal reproducible transcriptional structure in high-dimensional bulk RNA-seq settings.

From a practical perspective, several best practices emerge for AI-based bulk transcriptomic modeling. Feature selection should be performed exclusively within training data, for example, through nested selection within cross-validation, in order to prevent information leakage. Whenever independent datasets are available, external validation should be used to evaluate model generalization. In addition, model complexity should be matched to the available sample size and the number of selected features. Adhering to these principles can substantially reduce overfitting and improve the reproducibility of transcriptomic predictive models.

Future methodological development may further extend statistically guided feature selection frameworks to emerging transcriptomic modalities and integrative AI workflows. Increasingly, transcriptomic studies combine multiple molecular data types, including epigenomic, proteomic, and clinical information, which creates opportunities for multimodal AI models that integrate heterogeneous biological signals. In such settings, statistically grounded gene prioritization may provide a stable foundation for selecting informative transcriptomic features before multimodal integration. In parallel, advances in single-cell RNA-seq and spatial transcriptomics are generating datasets that capture cellular heterogeneity and tissue organization with unprecedented resolution. Although these technologies differ from bulk RNA-seq in structure and scale, they present similar challenges related to high dimensionality and noise. Feature prioritization strategies that account for the statistical properties of count-based expression data may therefore remain valuable components of analysis pipelines in these emerging contexts. Integrating statistically guided feature selection with multimodal AI frameworks and next-generation transcriptomic technologies represents a promising direction for improving interpretability, robustness, and biological insight in future computational genomics studies.

## References

[1] Berisha V, Krantsevich C, Hahn PR, Hahn S, Dasarathy G, Turaga P, Liss J. Digital medicine and the curse of dimensionality. NPJ Digit Med. 2021;4(1):153. 34711924 10.1038/s41746-021-00521-5 PMC8553745

[2] Crespo Márquez A. The curse of dimensionality. In: *Digital maintenance management: Guiding digital transformation in maintenance*. Cham (Switzerland): Springer; 2022. p. 67–86.

[3] Ying X. An overview of overfitting and its solutions. J Phys Conf Ser. 2019;1168: Article 022022.

[4] Rezapour M, Walker SJ, Ornelles DA, McNutt PM, Atala A, Gurcan MN. Analysis of gene expression dynamics and differential expression in viral infections using generalized linear models and quasi-likelihood methods. Front Microbiol. 2024;15:1342328. 38655085 10.3389/fmicb.2024.1342328 PMC11037428

[5] Rezapour M, Wesolowski R, Gurcan MN. Identifying key genes involved in axillary lymph node metastasis in breast cancer using advanced RNA-Seq analysis: A methodological approach with GLMQL and MAS. Int J Mol Sci. 2024;25(13):7306. 39000413 10.3390/ijms25137306 PMC11242629

[6] Rezapour M, Narayanan A, Gurcan MN. Machine learning analysis of RNA-seq data identifies key gene signatures and pathways in Mpox virus-induced gastrointestinal complications using colon organoid models. Int J Mol Sci. 2024;25(20):11142. 39456924 10.3390/ijms252011142 PMC11508207

[7] Rezapour M, Narayanan A, Mowery WH, Gurcan MN. Assessing concordance between RNA-Seq and NanoString technologies in Ebola-infected nonhuman primates using machine learning. BMC Genomics. 2025;26(1):358. 40211167 10.1186/s12864-025-11553-6 PMC11983957

[8] Rezapour M, Murphy SV, Ornelles DA, McNutt PM, Atala A. Tracing the evolutionary pathway of SARS-CoV-2 through RNA sequencing analysis. Sci Rep. 2025;15(1):23961. 40615624 10.1038/s41598-025-09911-1 PMC12227573

[9] Rezapour M, Bowser J, Richardson C, Gurcan MN. Transcriptional consequences of MeCP2 knockdown and overexpression in mouse primary cortical neurons. Int J Mol Sci. 2025;26(18):9032. 41009596 10.3390/ijms26189032 PMC12469642

[10] Rezapour M, McNutt PM, Ornelles DA, Walker SJ, Murphy SV, Atala A, Gurcan MN. Cross-modal predictive modeling of multi-omic data in 3D airway organ tissue equivalents during viral infection. Front Genet. 2025;16:1658577. 41070224 10.3389/fgene.2025.1658577 PMC12507369

[11] Robinson MD, McCarthy DJ, Smyth GK. edgeR: A Bioconductor package for differential expression analysis of digital gene expression data. Bioinformatics. 2010;26(1):139–140. 19910308 10.1093/bioinformatics/btp616 PMC2796818

[12] Del Giudice M, Peirone S, Perrone S, Priante F, Varese F, Tirtei E, Fagioli F, Cereda M. Artificial intelligence in bulk and single-cell RNA-sequencing data to foster precision oncology. Int J Mol Sci. 2021;22(9). 10.3390/ijms22094563 PMC8123853 33925407

[13] Zhou Z-H. *Ensemble methods: Foundations and algorithms*. Boca Raton (FL): CRC Press; 2025.

[14] Miikkulainen R, Liang J, Meyerson E, Rawal A, Fink D, Francon O, Raju B, Shahrzad H, Navruzyan A, Duffy N, et al. Evolving deep neural networks. In: *Artificial intelligence in the age of neural networks and brain computing*. Amsterdam (Netherlands): Elsevier; 2024. p. 269–287.

[15] Love MI, Huber W, Anders S. Moderated estimation of fold change and dispersion for RNA-seq data with DESeq2. Genome Biol. 2014;15(12):550. 25516281 10.1186/s13059-014-0550-8 PMC4302049

[16] Law CW, Chen Y, Shi W, Smyth GK. voom: Precision weights unlock linear model analysis tools for RNA-seq read counts. Genome Biol. 2014;15(2):R29. 24485249 10.1186/gb-2014-15-2-r29 PMC4053721

[17] Sun L, Wang T, Ding W, Xu J, Lin Y. Feature selection using Fisher score and multilabel neighborhood rough sets for multilabel classification. Inf Sci. 2021;578:887–912.

[18] Peng H, Long F, Ding C. Feature selection based on mutual information criteria of max-dependency, max-relevance, and min-redundancy. IEEE Trans Patt Anal Mach Intell. 2005;27(8):1226–1238. 10.1109/TPAMI.2005.159 16119262

[19] Tibshirani R. Regression shrinkage and selection via the lasso. J R Stat Soc Series B Stat Methodol. 1996;58(1):267–288.

[20] Zou H, Hastie T. Regularization and variable selection via the elastic net. J R Stat Soc Series B Stat Methodol. 2005;67(2):301–320.

[21] Kursa MB, Rudnicki WR. Feature selection with the Boruta package. J Stat Softw. 2010;36:1–13.

[22] Meinshausen N, Bühlmann P. Stability selection. J R Stat Soc Series B Stat Methodol. 2010;72(4):417–473.

[23] George EI, McCulloch RE. Variable selection via Gibbs sampling. J Am Stat Assoc. 1993;88(423):881–889.

[24] Abdi H, Williams LJ. Principal component analysis. WIREs Comput Stat. 2010;2(4):433–459.

[25] Lee T-W. Independent component analysis. In: *Independent component analysis: Theory and applications*. Boston (MA): Springer; 1998. p. 27–66.

[26] Hinton GE, Salakhutdinov RR. Reducing the dimensionality of data with neural networks. Science. 2006;313(5786):504–507. 16873662 10.1126/science.1127647

[27] Bengio Y, Courville A, Vincent P. Representation learning: A review and new perspectives. IEEE Trans Pattern Anal Mach Intell. 2013;35(8):1798–1828. 23787338 10.1109/TPAMI.2013.50

[28] Srivastava N, Hinton G, Krizhevsky A, Sutskever I, Salakhutdinov R. Dropout: A simple way to prevent neural networks from overfitting. J Mach Learn Res. 2014;15(1):1929–1958.

[29] Loshchilov I, Hutter F. Decoupled weight decay regularization*.* arXiv preprint arXiv. 2017 10.48550/arXiv.1711.05101

[30] Anders S, Huber W. Differential expression analysis for sequence count data. Genome Biol. 2010;10(10):R106. 10.1186/gb-2010-11-10-r106 PMC3218662 20979621

[31] Robinson MD, Oshlack A. A scaling normalization method for differential expression analysis of RNA-seq data. Genome Biol. 2010;11(3):R25. 20196867 10.1186/gb-2010-11-3-r25 PMC2864565

[32] Chen Y, Lun AT, Smyth GK. From reads to genes to pathways: Differential expression analysis of RNA-Seq experiments using Rsubread and the edgeR quasi-likelihood pipeline. F1000Res. 2016;5:1438. 27508061 10.12688/f1000research.8987.1 PMC4934518

[33] Benjamini Y, Hochberg Y. Controlling the false discovery rate: A practical and powerful approach to multiple testing. J R Stat Soc Ser B Stat Methodol. 1995;57(1):289–300.

[34] Rezapour M, Walker SJ, Ornelles DA, Niazi MKK, Mcnutt PM, Atala A, Gurcan MN. Exploring the host response in infected lung organoids using NanoString technology: A statistical analysis of gene expression data. PLOS ONE. 2024;19(11): Article e0308849. 39591472 10.1371/journal.pone.0308849 PMC11594423

[35] Rezapour M, Niazi MKK, Lu H, Narayanan A, Gurcan MN. Machine learning-based analysis of Ebola virus’ impact on gene expression in nonhuman primates. Front Artif Intell. 2024;7:1405332. 39282474 10.3389/frai.2024.1405332 PMC11392916

[36] Hilbe JM. *Logistic regression models*. Chapman and Hall/CRC; 2009.

[37] Hearst MA, Dumais ST, Osuna E, Platt J, Scholkopf B. Support vector machines. IEEE Intell Syst Appl. 1998;13(4):18–28.

[38] Ogutu JO, Piepho H-P, Schulz-Streeck T. A comparison of random forests, boosting and support vector machines for genomic selection. BMC Proc. 2011;5 Suppl 3(Suppl 3):S11. 10.1186/1753-6561-5-S3-S11 PMC3103196 21624167

[39] Rezapour M, Opoku LA, Trefry SV, Alili A, Konadu M, Dionsio MG, Gurcan MN, Narayanan A. Transcriptomic profiling of human endothelial cells infected with Venezuelan equine encephalitis virus reveals NRF2 driven host reprogramming mediated by Omaveloxolone treatment. Front Genet. 2025;16:1722527. 41480150 10.3389/fgene.2025.1722527 PMC12755857

